# Palaearctic Egg Parasitoids Interaction to Three Grapevine Exotic Pests in Northwestern Italy: A New Association Involving *Metcalfa pruinosa*

**DOI:** 10.3390/insects11090610

**Published:** 2020-09-08

**Authors:** Federico Marco Bocca, Luca Picciau, Stefania Laudonia, Alberto Alma

**Affiliations:** 1 Department of Agricultural, Forest and Food Sciences and Technologies, University of Turin, Largo P. Braccini 2, 10095 Grugliasco (TO), Italy; federicomarco.bocca@unito.it (F.M.B.); luca.picciau@unito.it (L.P.); 2 Department of Agricultural Sciences, University of Naples Federico II, Via Università, 100, 80055 Portici (NA), Italy; laudonia@unina.it

**Keywords:** parasitization rate, host density, vegetation cover, flatid, *Scaphoideus titanus*, *Orientus ishidae*, *Neodryinus typhlocybae*, vineyard agroecosystem, biological control

## Abstract

**Simple Summary:**

*Scaphoideus titanus*, *Orientus ishidae* and *Metcalfa pruinosa* are exotic hoppers considered as the most important pest for Italian viticulture. They are usually controlled by insecticides in industrial viticulture. These pest populations tend to increase in uncultivated areas within the vineyard agroecosystem where the pesticides are forbidden. Therefore, we investigated the presence of egg parasitoids, as possible biocontrol agents, and the interaction with other parasitoids in these environments. No specimen emerged from the eggs of the first two leafhoppers, while many specimens identified as *Oligosita collina* group emerged from the eggs of the planthopper *M. pruinosa*, which represents a new association. We consequently evaluated its parasitization rate, which showed a very high value, in different Piedmontese wine growing areas. The preliminary study pointed out a higher percentage of the egg parasitoid compared to the currently used biocontrol agent *Neodryinus typhlocybae*. The identification of an oophagous able to adapt to this flatid open up new perspective on *M. pruinosa* control.

**Abstract:**

The most important exotic leafhopper pests currently affecting the Italian vineyards are the leafhoppers *Scaphoideus titanus*, *Orientus ishidae* and the planthopper *Metcalfa pruinosa*. Their highest population density is detected in the uncultivated areas with wild grapevines. Should these habitats be considered only a problem or a potential resource for Palearctic entomophagy of these three exotic pests? The aim of this work was to study the biotopes and biocoenosis present in the Piedmontese vineyard agroecosystem, evaluating the parasitization rate and other crucial aspects for a possible application in biological control. Several specimens of egg-parasitoid wasps were obtained from filed-collected two-year-old grapevine canes. The most prevalent one belonged to the *Oligosita collina* group (Trichogrammatidae) emerged only from *M. pruinosa* eggs with a parasitization rate of over 40%. The new association is the first report of such a high level of parasitization on the flatid planthopper. The parasitization rate mainly relied on the host egg density and the abundance of plants suitable for the oviposition. A second parasitoid generation on the overwintering eggs is discussed, as well as other hypothesis. Furthermore, the parasitization rate was higher than the one showed by the dryinid *Neodryinus typhlocybae*, the control agent introduced in Italy under the biological control strategy, highlighting a possible implication in this biocoenosis. We assume that the egg parasitoid adaptation may contribute to *M. pruinosa* control.

## 1. Introduction

Some of the most important grapevine pests in Italy are exotic and have been introduced at different times. In particular, concerning the order Hemiptera suborder Auchenorrhyncha, *Scaphoideus titanus* Ball (Membracoidea: Cicadellidae: Deltocephalinae), a Nearctic species known as the main vector of 16SrV phytoplasmas agents of Flavescence dorée (FD) [1] was reported for the first time in Italy in 1963 [2]. Similarly, *Metcalfa pruinosa* Say (Fulgoroidea: Flatidae) was introduced from North America in 1979 [3]. Its strong infestations cause economic and aesthetic damages due to the abundant production of honeydew associated with epiphytic fungi [4]. *Orientus ishidae* Matsumura (Cicadellidae: Deltocephalinae), native of Asia, was reported as an alien species Italy in 1998 [5], and recently recognized as a putative vector of the FD [6].

*Scaphoideus titanus* is univoltine species and specialist of the grapevine (*Vitis* spp.) [7]. The eggs are laid under the bark of at least a two-year old wood, where they overwinter. Adults arise from mid-June until mid-October [8]. Recently, new insights on some biological traits have demonstrated that longevity and fecundity were largely underestimated in literature. In fact, males can live over 40 days, whilst females over 60 days and lay an average of about 60–65 eggs [9].

The planthopper *M. pruinosa* is a univoltine species that overwinters as eggs, and has a gregarious behavior. It is able to feed on a wide range of plants [4], and lay an average of 60 eggs [10]. Eggs are laid under the bark from August, hatching takes place from May till July, and adults emerge in the middle of July or early August [3,4]. In some geographical areas it has been recently reported as responsible for considerable damages to crops, forest and ornamental trees [11,12,13,14]. Moreover, new information emerged on its ability to transmit phytoplasmas belonging to the subgroup 16SrI-B ‘*Candidatus* Phytoplasma asteris’, agents of the Aster yellow disease [15].

*Orientus ishidae* has one generation per year and overwinters as eggs [16]. Adults live on different host plants, such as hazelnut, hornbeam, willow, and others, often present in the surrounding of the vineyards [6]. It is highly polyphagous and can lay eggs on grapevines even if in the nymphs, which can be found from the middle of May up to the middle of July, feed on other plants rather than grapevine, and adults emerge from mid-May until mid-September [17].

The highest population density of these three species can be detected in the ecological corridors formed by uncultivated areas or woods with wild grapevine, representing a source of re-infestation flows [4,17,18]. In such environments, indeed, insecticides are not allowed since they would have serious negative repercussions on the present biocoenosis. The absence of effective control strategies against FD-vector species spread raises the need to consider such environments as an opportunity, rather than a mere issue. In fact, they can represent the ideal habitat for native predator/parasitoids that can limit these three pests. The egg is the most vulnerable stage for leafhoppers [19], and egg parasitoids are considered the most important natural enemies of Auchenorrhyncha [20,21]. In particular, since these species spend a large part of their cycle at the egg stage, it would be essential to identify any egg parasitoids able to adapt to them.

Nevertheless, limited information is currently available on egg parasitoids adapted to these exotic species. Concerning *S. titanus*, in North America, some oophagous, never identified at a specific level, were sporadically reported: Hymenoptera Mymaridae and Trichogrammatidae [22]. Likewise, in Italy, some parasitoids from *S. titanus* eggs were obtained, but also, in this case, the parasitization rates were modest (<1%) [23]. Additionally, concerning egg parasitoids of *M. pruinosa,* scattered notes have been reported in literature. In North America, only a species of *Telenomus* emerged from its eggs [24] while in Italy only some specimens of *Centrodora livens* (Walker) (Hymenoptera: Aphelinidae) have been observed [25]. In all cases, the parasitization rate was too low for possible use in biological control (<1%). Unlike the other two exotic pests, *M. pruinosa* has been the subject of an important biological control strategy in Italy since 1987, with the introduction of *Neodryinus typhlocybae* (Ashmead) (Hymenoptera: Dryinidae) from North America [26], which attacks only the juvenile instars accomplishing two generations per year. The dryinid has had a key role in its control [4,27,28,29] and has been able to follow its host in other countries without a direct introduction [30,31]. Regarding *O. ishidae*, to date hardly any information is available in the literature on egg parasitoids that are able to adapt on it.

One of many important factors that could affect the egg parasitoids activity in the field is the host density and its interaction with the ecological context [32,33,34]. Foraging parasitoids tend to prefer areas where their hosts are present at a higher density and adapt their foraging behavior to host density variation [35,36,37]. Nevertheless, in some cases the parasitization rate can be independent or inversely-dependent from the host density [32,33,38], even if a recent meta-analysis suggested that positive spatial density dependence is more common than a negative correlation [34]. Habitat complexity and its interaction with the host density is another factor which could drive the field parasitization rate [39,40,41]. As far as the egg parasitoids of plant-leafhoppers are concerned, conflicting results regarding the dependence on host density according to different spatial scales and different patches are reported [42,43,44].

The aim of this work is to study the biotopes and biocoenosis present in the vineyard agroecosystem. Furthermore, we pay particular attention to a possible Palaearctic egg parasitoid adaptation to these three exotic species and the possible interaction with other parasitoids. The parasitization rate, in percentage, on different plant species and some variables such as the host egg density and the vegetation cover are evaluated as well as other crucial biological aspects for a possible application in the biological control strategy.

## 2. Materials and Methods

This research was carried out over three years: during the first year an extended survey was performed by means of sentinel eggs to assess the possible entomophagous adaptation to the three pests. Based on these results, in the following two years an experimental design in two important areas of the Piedmontese vineyard agroecosystem was set up to evaluate the parasitization rate of the parasitoid identified from *M. pruinosa* and the possible influence on the parasitization rate of plant species, host egg density and plant cover. During the same years we carried on preliminary studies upon the voltinism of the egg parasitoid and its interaction with *N. typhlocybae*.

### 2.1. Survey on Metcalfa pruinosa, Scaphoideus titanus and Orientus ishidae Egg Parasitoids Complex and Host Parasitization Specificity

One and two-year-old canes of grapevine, *Corylus avellana* L., *Ulmus* spp., *Acer* spp., *Cornus sanguinea* L. and *Sambucus nigra* L. were collected from brushwood in three Piedmontese grapevine growing areas. The collection was carried out in 20 localities of Torino, Asti and Cuneo provinces during winter 2016. Portions of vine shoots were inspected by gently removing the bark by means of a lancet. Then, cane sections hosting eggs were incubated at 25 °C in falcon tubes, provided with damp cotton-wool and closed with an insect-proof-net.

During the summer of 2017 sleeve-cages (1.6 mm × 1.6 mm, to allow egg parasitoids to enter and prevent the leafhoppers from escaping), containing vine canes sterilized in an autoclave and living leafhoppers (ten females of one species in each cage) were placed in Piedmontese viticulture areas where the three mentioned pests were present at high population density and several oophagous were found the previous year, to verify the specificity of the egg parasitoids. Five cages for each species, for a total of 15, were placed in every area. At the end of the winter, the canes were incubated at 25 °C in falcon tubes, provided with damp cotton-wool and closed with the insect-proof net.

### 2.2. Parasitization Rate, Voltinism and Emergence Curves

In order to investigate the difference in terms of egg-parasitization rate on *M. pruinosa* among areas and years, a survey was conducted on the three most common plant species present in the vineyard agroecosystem of two Piedmontese areas, located in the provinces of Asti (Area 1) and Cuneo (Area 2) (see Figure S1 and Table S1 in Supplementary Material). These environments are characterized by agricultural lands, with a high density of vineyards and uncultivated areas or brushwood with wild grapevine. Five sites per area were randomly chosen in uncultivated areas, woods with wild grapevine or abandoned vineyards. For each site a surface of about 1500 m² extent was sampled subdividing each site in sampling units. Five kilograms of two-year-old grapevine canes (*Vitis* spp.), hazelnut (*C. avellana*) tree and dogwood (*Cornus* spp.) were randomly collected during the winters of 2017 and 2018. Portions of twigs from each plant species were inspected, to sort out the eggs of each pest or the canes containing eggs, and then incubated as previously described. In each site the host egg density per plant species was calculated as the total number of eggs (parasitized + not parasitized) counted for the total amount of woods per plant species collected in each site, divided by the total number of the same plant species. The influence of the habitat complexity on the parasitization rate, interpreted as the homogeneous diffusion of plants suitable for *M. pruinosa* oviposition, in each site was evaluated considering the vegetation cover percentage of the three aforementioned plant species. The plant cover was quantified as the number of the plant species (count) divided by the total number of trees present in each site.

In order to evaluate voltinism and emergence curves, approximately 500 parasitized eggs were preserved in falcon tubes provided with damp cotton wool and closed with the insect-proof net, under out-door condition conditions, in spring 2019. The tubes were constantly monitored; the parasitization rate and the flight curves were calculated. Moreover, 300 parasitized eggs of *M. pruinosa* were isolated and incubated at 25 °C to verify the number of egg-parasitoid emerging from each egg. 

### 2.3. Comparison between the Parasitization Rate of Neodryinus Typhlocybae and Oligosita Collina Group on Metcalfa pruinosa

The study was carried out in eight sites in Turin province during 2019 (see Figure S1 and Table S2 in Supplementary Material). Each site was subdivided into subunits according to a stratified sample. The nymphs were counted for a length of time of 5 min in each subunit. During the previous winter two-year-old grapevine canes, and twigs of other tree species were randomly collected in each subunit for an amount of 10 kilos of wood per site. Cane sections hosting eggs were investigated and incubated as previously described. The parasitization rate of *N. typhlocybae* in each site and each subunit was calculated as follow: a first count on first generation cocoon at the end of July when the species shows its peak in northern Italy [4], and a second count on the overwintering cocoons at the beginning of September.

### 2.4. Statistical Analysis

Data analyses were conducted in R version 3.5.2 (R Core Team 2018). *M. pruinosa* eggs parasitization was analyzed by Generalized linear mixed model (GLMM) with a logit-link and assuming a logistic distribution for the error of the latent variable. The analysis was conducted using the glmer function in the lme4 package in R [45]. The response, originally recorded as parasitization success or failure, was linearly modelled using the log odds transformation. The fixed effects included in this model were Plant Species, Host Eggs Density, Plant Cover and Year together with some interaction between them. Specifically, the interaction between Plant Species and Plant Cover, between Plant Species and Host Eggs Density, between Year and Plant Species, and finally among Plant Species, Host Eggs Density and Plant Cover were considered Year (two levels), Plant Species (*Vitis* spp., *C. avellana*, *Cornus* spp.) were modelled as categorical variables whilst standardized Plant Cover and standardized Host Eggs Density were used as continuous variables. GLMM was fitted using Site nested within Area as random effects to take into account the dependencies among measures in each Area. The analysis unit of the Host Egg Density calculated in this survey, represents an aggregated data for each site (the sampling took place on a specific quantity of wood collected per site, but not on a specific number of plants determined in advance.) Therefore, the spatial scale (*sensu* Dungan [46]) of host density was not comparable to a grain size of plant but rather to a larger grain size like the site scale. Furthermore, the effect on area extent could not be calculated since areas were too large to be treated as patch [32].

The best subset selection method was used to select the best model for this purpose: all possible models are compared using a subset of aforementioned predictors [47]. Log-likelihood ratio tests (LRTs) [48] and the Akaike’s information criterion (AIC) were used to evaluate the candidate models, the selected model was chosen also according to the best results interpretability.

Based on the results of the chosen model, a further model to inspect the influence of the variables Year, Plant Species and Plant Cover on *M. pruinosa* oviposition was proposed. Therefore, a GLMM with a negative binomial distribution for the response was performed using the glmer.nb function in the lme4 package in R. This distribution was preferred over other models based on the comparison of the AIC values. The response variable was number of *M. pruinosa* eggs per plant in each site. The model included Year (two levels), Plant Species (three levels), Plant Cover (continuous variable) and the interaction between Plant Species and Plant Cover and between Plant Species and Year as fixed effects; while Site nested to Area as a random effect. In order to consider the number of plants on which the wooden pieces were collected, an offset variable was included. As described for the previous model, the model was chosen by means the best subset selection method.

An LRT test was used to verify the significance of the fixed effects and the overall significance of the model, comparing the fitting model to “null” models (including only the random effects). Full model results and overall significance tests for models are presented in Supplementary Material.

For the selected model chosen by AIC, the residuals were examined using the function testUniformity() from the DHARMa package [49] (see Figure S3 in Supplementary Material). Marginal means and contrast were also estimated using the emmeans package (EMMs) [50].

Concerning the survey on *N. typhlocybae* and *O*. cf *collina* parasitization, three variables have been taken into account: the number of *M. pruinosa* eggs/nymphs parasitized and not parasitized; Parasitoid Species (two levels) and Site (eight levels). Two hypotheses were tested: marginal independence, namely if Parasitization and Species are independent; conditional independence: if their relationship can be explained by Site, specifically if the independence of the variables Parasitization and Species exists, given the variable Site. The hypothesis of marginal independence between Species and Parasitization variables ignoring the Site variable was verified using the chi-square (ꭕ^2^) test. The Cochran-Mantel-Haenszel test for 8 partial 2 × 2 contingency tables controlled for Site was performed to verify the independence of the Parasitization and Species variables given by the Site variable (conditional independence model).

## 3. Results

### 3.1. Survey on Metcalfa pruinosa, Scaphoideus titanus and Orientus ishidae Egg Parasitoids Complex and Host Parasitization Specificity

During 2017 a total of 343 *M. pruinosa*, 746 *S. titanus* and 134 *O. ishidae* nymphs were counted. From the incubation of parasitized eggs of *M. pruinosa,* two species of egg parasitoids emerged: a total of 191 females and 157 males which belonged to *Oligosita collina* group (Hymenoptera: Trichogrammatidae) (Laudonia, *in litteris*) (*sex ratio* 1:0.9; females to males) hereafter named as *O.* cf *collina*, and 127 females of *Centrodora livens* (Walker) (Hymenoptera: Aphelinidae). No egg parasitoids emerged from the incubation of the eggs of *S. titanus* and *O. ishidae*. Only two old apparently parasitized *S. titanus* eggs, each one with a jagged hole, were observed during the inspection of grapevine canes. 

As regards to the host parasitization specificity trial, in 70% of sleeve-cages with *M. pruinosa* eggs emerged specimens of *O.* cf *collina* while no specimens of *C. livens* were observed. No egg parasitoids emerged from canes with eggs of *S. titanus* and *O. ishidae*.

### 3.2. Parasitisation Rate, Voltinism and Emergence Curves

During winter 2017/2018 the parasitization rate was 41% ± 6.05 (mean ± standard deviation) in Area1 (median = 41%; interquartile range IQR, 40–44; *sex ratio* 1:1.4), 44.6% ± 5.80 in Area 2 (median = 46%; IQR, 44–48; *sex ratio* 1:1.9). During winter 2018/2019 the parasitisation rate was 39 % ± 19.3 in Area1 (median = 40%; IQR, 37–53; *sex ratio* 1:1.6), 36.7% ± 17.3 in Area 2 (median = 39%; IQR, 20–47; *sex ratio* 1:1.2).

#### 3.2.1. *Metcalfa pruinosa* Eggs Parasitization Model

The selected model included the fixed predictors Host Egg Density, Plant Cover, Plant Species and the interaction between Host Egg Density and Plant Species. While no significant differences (using alpha < 0.05) in the parasitization percentage concerning the predictor Year, the interaction between Year and Plant Species, the interaction between Plant Species and Plant Cover, and the interaction among Plant Species, Plant Cover and Host Egg Density were found (see Tables S3 and S4 in Supplementary Material). Therefore, the latter predictors were dropped from the chosen model. The selected model featured the best residual diagnostic among the top five models of the best subset selection. This model indicated that when the factor Plant species referred to *Cornus* and the value is 0 for the factors Host Egg Density and Plant Cover, the baseline odds of the parasitization rate is 0.34. The baseline odds decrease by 25% when the Plant Species is *Corylus* and increase by 30% when the Plant species is *Vitis.* All else being equal, a unit change in Host Egg Density is associated with a 68% increase in the odds of being parasitized. *Vice versa*, when the value of Host Egg Density is 0, a unit change in Plant Cover is associated with a 33% increase in the odds of being parasitized. The interaction term shows that the odds ratio of Host Egg Density was estimated to increase by a multiplicative factor of 1.11 for each extra unit of Plant Cover, with an increment of 88% in the odds of being parasitized.

**Figure 1:**
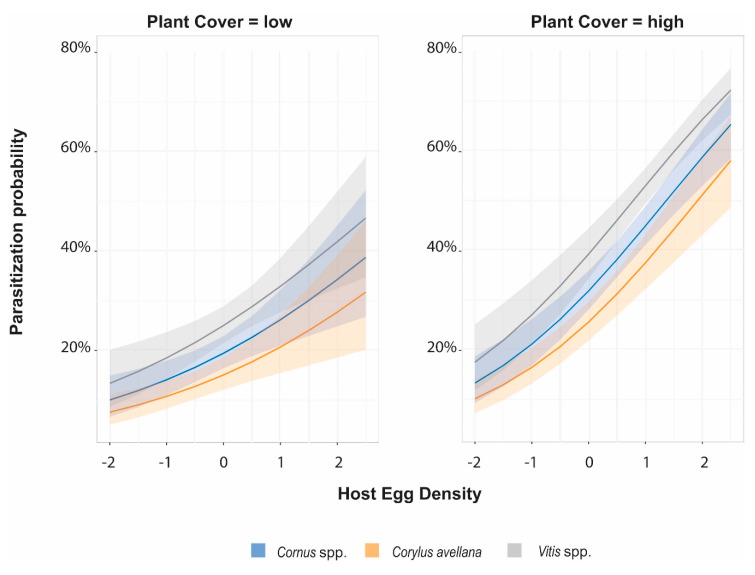
Predicted probability of egg parasitization for three Plant Species, with respect to standardized Plant Cover and standardized Host Egg Density (number of *M. pruinosa* eggs per Plant Species in each site). The percentage of Plant Cover was modeled as continuous variable. Two representative values of high and low level of the standardized distribution depicted by one standard deviation below and above mean value are reported in this graph. Lines represent the logistic fit, shading the 95% confidence interval. The probability of being parasitized increased with either the host density and the Plant Cover. Model coefficients are provided in the Supplementary Material.

From the Figure 1 one might suppose that the parasitization rate at high values of Host Egg Density was greater at higher levels of Plant Cover than at lower levels for all plant species. Whilst it might be deduced that in correspondence with the minimum values of the Host Egg Density both at higher and lower levels of Plant Cover, no differences in the odds of parasitization rate between different Plant Species existed.

**Figure 2:**
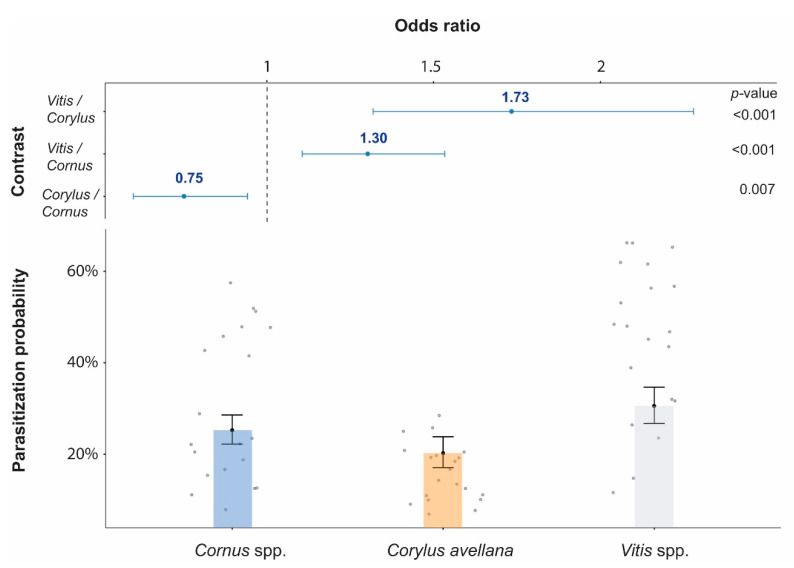
Top: the y-axis shows all pairwise comparisons among the three Plant Species levels, while the x-axis represents the response which is the Odds ratio of the means of the two groups of Plant Species in the comparison (*p*-values from the hypothesis tests are included on the right). Bottom: the estimated marginal means of the parasitization probability on the three plant species are represented. Data are presented on the back-logit scale with 95% confidence intervals (CI). The raw data are plotted as grey dots. Model coefficients are provided in the Supplementary Material.

The pairwise comparisons show that the marginal means of the parasitization rate was higher on grapevine compared to the other two plant species, and in *Cornus* was higher than *Corylus* (Figure 2). 

#### 3.2.2. *Metcalfa pruinosa* Oviposition Model

Concerning the model of *M. pruinosa* oviposition rate among plants, the final best-fit model included only the fixed factors Plant species and Plant cover whilst their interaction was not a significant predictor, and was dropped from the final selected model (see Tables S5 and S6 in Supplementary Material). When the factor Plant species referred to *Cornus* and the value is 0 for the factor Plant cover, the baseline rate of the oviposition is 8.1. It decreases by 31% when the Plant Species is *Corylus* and increase by 32% when the Plant Species is *Vitis.* All else being equal, a unit change in Plant Cover is associated with 25% increase in the rate of oviposition.

**Figure 3:**
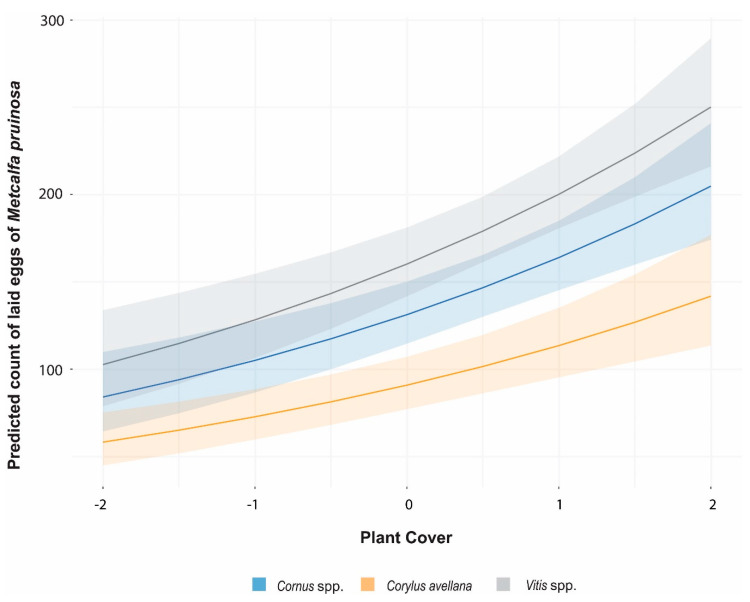
Curves of *Metcalfa pruinosa* oviposition on three Plant Species as a function of the standardized variable Plant Cover. Lines represent the Poisson negative binomial fit, shading represents the 95% confidence interval. The oviposition rate increased with Plant cover but differently according to the Plant Species.

**Figure 4:**
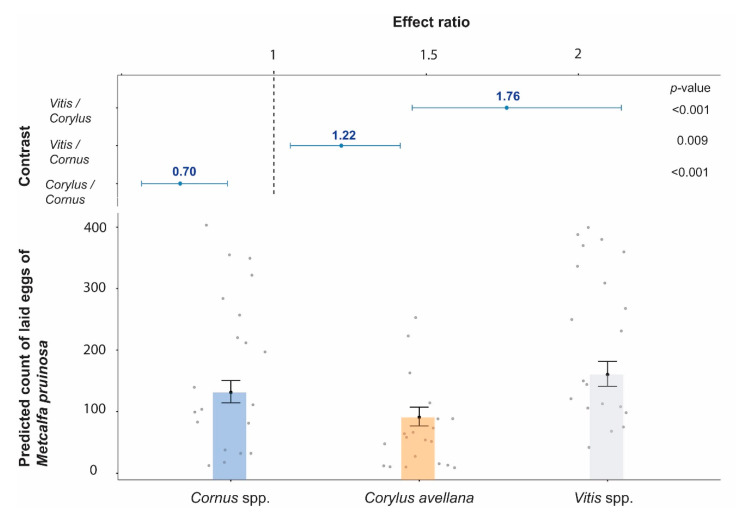
Top: the y-axis shows all pairwise comparisons among the three Plant Species levels, while the x-axis represents the response which is the ratio of the means of the two groups of Plant Species in the comparison (*p*-values from the hypothesis tests are included on the right). Bottom: the estimated marginal means of *Metcalfa pruinosa* oviposition on the three Plant Species. Data are presented on the back-Poisson negative binomial scale with 95% confidence intervals (CI). Model coefficients are provided in the Supplementary Material.

The pairwise comparisons show that the oviposition rate of *M. pruinosa* was greater on grapevine than the other plants, whilst in *Cornus* was higher than *Corylus* (Figure 3 and Figure 4)

These models show that the parasitization depends on the number of eggs laid by *M. pruinosa*, which varies on different plant species.

#### 3.2.3. Voltinism and Emergence Curves

Concerning the *O.* cf *collina* flight curves, in 2019 two peaks during the season were observed. The first specimen was collected on 24 April with the highest peak on 15 May and then ended 10 June. The second curve began 19 July with the highest peak on 27 July and ended on 8 August (see Figure S2 in Supplementary Material).

### 3.3. Comparison of the Parasitisation Rate of Neodryinus typhlocybae and Oligosita cf collina on Metcalfa pruinosa

**Figure 5:**
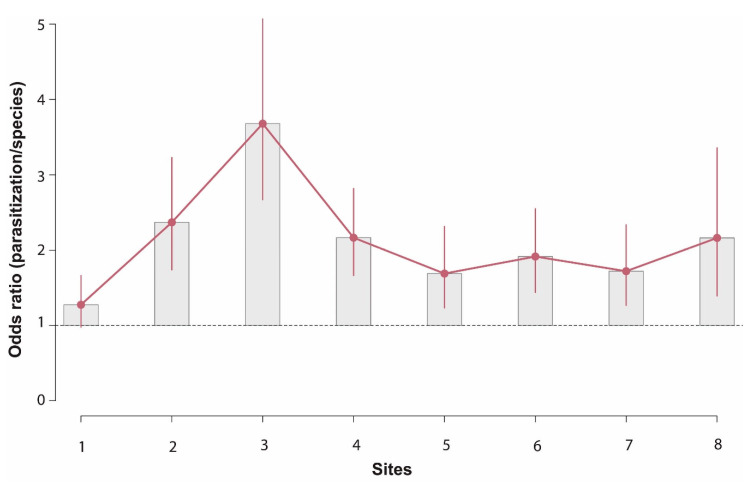
Odds ratios for parasitization and sites. Data are presented with 95% confidence intervals (CI). The highest value of odds ratio is found in site 3, where the odds of being parasitized by *Oligosita* cf *collina* are 3.68 times higher than *Neodryinus typhlocybae*. Only in site 1, where the odds of parasitization by the egg parasitoid increase of 27% compared to dryinid, the comparison was not significant.

The odds are significantly higher for *O.* cf *collina* than *N. typhlocybae* in seven sites out of eight (Figure 5). The parasitization rate of *N. typhlocybae* ranged from 18% to 32% (see Table S7 in Supplementary Material). As regard the hypothesis of marginal independence of parasitoid species and *M. pruinosa* parasitization rate, the null hypothesis is refused using reasonable levels of significance (*ꭕ*^2^ = 168.7 *df* = 1, *p*-value < 0.0001). The odds of parasitization *O.* cf *collina* are 2.01 (95% CI 1.81–2.23) times as high as *N. typhlocybae*. This relationship cannot be explained by the influence of the variable sites, in fact, parasitoid species and parasitization rate are not independent to the given sites (Mantel-Haenszel *ꭕ*^2^ = 160.75 *df* = 1, *p*-value < 0.0001). Mantel-Haenszel estimate of the common odds ratio is 1.98 (95% CI 1.78, 2.21).

## 4. Discussion

Insect parasitoids spend most of their limited time in searching for a suitable host to reproduce. This activity is particularly arduous for egg-parasitoids since their immobile, and often hidden hosts are hard to be located from a distance [51,52,53]. Egg-parasitoids have evolved the ability to detect host’s volatile signals [54,55,56], and usually show a wide host range [57,58,59].The presence of a new exotic potential host can lead to novel interactions and adaptation [60,61].

Our results highlight that no egg parasitoids emerged from the rearing of *S. titanus* eggs as well as of *O. ishidae* eggs. Some authors had sporadically observed egg parasitoids able to parasitize eggs of *S. titanus*, with a low parasitization rate [22,23]. The only two apparently parasitized eggs observed during this research, allow us to confirm those results and state that the *S. titanus* egg parasitization is uncommon. As far as *O. ishidae* is concerned, it is worthwhile to notice that fewer eggs of this leafhopper are found compared to the other two pests. Besides, it is known that *O. ishidae* lays eggs on grapevine in considerably lower number compared to other plants [17], and the grapevine was the most abundant species plant in the vineyard agroecosystem. Therefore, we cannot state with certainty that no egg parasitoids were able to adapt on this leafhopper.

In regard to *M. pruinosa*, at least two species of egg parasitoids have adapted to the flatid, that is *O.* cf *collina* and *C. livens* with a clear predominance of the former. The latter was reported as *M. pruinosa* eggs parasitoid in a previous study without a specific parasitization rate [25,58].The genus *Centrodora* Forester is considered cosmopolitan and includes primary and secondary parasitoids which develop on insects belonging to different orders [62].

Many genera belonging to the sub-family Oligositinae are known to parasitize insects belonging to different orders including Diptera, Coleoptera, Orthoptera and Cicadellidae [63,64,65]. The genus *Oligosita* Walker was redescribed by Pinto and Viggiani [63]. Lately, the systematic contribution in describing new *Oligosita* species has increased [66,67,68,69], however, for *O. collina* group, as defined by Nowicki [70] and Viggiani [71], many species need to be identified yet, and their taxonomic position and associations with hosts found out. The association here described represents a new biocoenosis involving the exotic planthopper and *O.* cf *collina*. Indeed, several studies, indirectly concerning the possible adaptation of egg parasitoids to *M. pruinosa,* have never observed such a high parasitization rate [24,25].

The knowledge of biological traits as voltinism and parasitization rate of genera non-*Trichogramma* within the Trichogrammatidae is still limited [65,72]. Maybe due to the different data collection methods, many other cases of egg parasitism among Oligositinae show different field parasitization rates. For instance, many species within Oligositinae were able to exert a high level parasitization rate on their hosts: the complex *Pseudoligosita aesopi* (Girault) and *Oligosita* *naias* Girault reached a maximum of 47% on *Nilaparvata lugens* (Stål) (Hemimptera: Delphacidae) [73], and *Paracentrobia tapajosae* Viggiani exhibited a parasitization rate of about 45% on *Dalbulus maidis* (DeLong and Wolcott) [74,75]. Other species of Oligositinae showed an interesting potential biological pest control [76,77,78]. In spite of these few examples, many Oligositinae species showed a negligible parasitization rate [73,74,79]. The parasitization rate of *O.* cf *collina* on *M. pruinosa* (an average of more than 40 % in the survey areas and over 50 % in some sites) is similar to the parasitoid complex with the highest level of parasitization, and being a new association, can be deemed remarkable. The parasitization rate was high in both years, but it is reasonable to expect a reduction of these values in consideration of the host-parasitoid dynamics trend of [80,81].

The high parasitization level exhibited by *O.* cf *collina* allows us to affirm that the egg parasitoids spread in Piedmont and that a stable association with *M. pruinosa* in this region exists. Even if further studies are needed to confirm it over the years. From the results of the models, it can be deduced how *O.* cf *collina* is able to parasitize *M. pruinosa* eggs on plants of different species. The parasitization rate is mainly related to the density of host eggs and the abundance of plants that can be used as a substrate of oviposition.

Regarding the attraction due to the density of the host eggs, some studies on plant-leafhoppers have reported a different density-dependent relationship between host density and parasitization rate [42,43,44]. However, the dynamics and the fitness factors behind this relation have not been proven yet. The preliminary results of this study reveal a positive effect of the host density on the parasitization rate. The host density calculated in this study refers to the density of eggs per plant species at a given site, while no multi-scale analysis was performed. Therefore, it is not comparable to a finer grain size like leaf as reported in some of the aforementioned studies. Moreover, Segoli [43] observed a positive dependence at a grain size called field scale. Our results, referring to a site grain size, are in agreement with this conclusion. According to some authors, if the density is evaluated at finer scales, the relationship with the parasitization tends to become zero or negative, since oviposition activity responds to other factors barely quantifiable and not fully correlated with fine-scale host density, and can increase with the observational grain size [32,33]. Nevertheless, in a multi-factor meta-analysis study, Gunton and Pöyry [34] found limited and ambivalent support for the scale-specific foraging hypothesis. More in-depth multi-scale studies are needed to clarify these aspects in this new biocoenosis on a finer grain size.

Concerning the reasons behind the positive dependence observed in this study, *O*. cf *collina* could be attracted by the exudates or honeydew produced by the *M. pruinosa* [82], as already known for other parasitoids [83]. This secretion is another essential factor that can act as a contact-kairomone as reported for several insects, including plant-leafhoppers [20,84,85]. As reported in previous studies, egg parasitoids belonging to other genera of Trichogrammatidae respond to volatile compounds produced by or associated with host eggs, including scales from adult moths, sex pheromones, and compounds related to egg metabolic processes [86,87,88].

*Mectalfa pruinosa* is polyphagous and might feed and oviposit on the most prevalent plant species. Therefore, on these plants, the oviposition rate is likely to be higher as well as the consequent parasitization rate. In this research, the parasitization rate was greater on grapevine, both in case of lower and higher plant cover, than on the other plant species. It could be assumed that this flatid behavior may be promoted by the characteristic bark of grapevine and by its growth habitus in the wood, even if this potential preference should be clarified and demonstrated. Nevertheless, in the absence of evidence pointing out the involvement of semiochemicals, we cannot exclude that the higher values of the parasitization rate on *Vitis* spp. is ascribable to synomones [89,90,91,92], as well as a major oviposition rate of *M. pruinosa* on the grapevine.

The complexity and fragmentation of the habitat can also influence the parasitization success and the host densities [39,41]. In our study, the interaction between host egg density and plant cover shows that a simplification of the landscape structure, meaning the presence of many plants with many host eggs instead of few plants with many eggs, can favor the parasitoid searching efficiency. This suggest that the host density alone does not explain the parasitization rate, but the activity of the oophagous can be favored by a structurally simple surface of the site [39], in our study represented by a solution of continuity between plants bearing the host eggs.

Another important factor in a biological control perspective concerns the number of generations that the parasitoid is able to perform on the host. Results on the voltinism trial reveal that the first adults emerge in the middle of April. During the adult emergence in April, no new host eggs were available while some *O*. cf *collina* specimens were collected at the time of the first host oviposition. This observation led us to hypothesize a second generation on *M. pruinosa* wintering eggs like *Epoligosita vera* Viggiani, which accomplishes two generations on the overwintering eggs of *Lindbergina aurovittata* (Douglas) [78]. If this is true, *O*. cf *collina* would accomplish its second generation onto the same wintering eggs and benefit from not being forced to move and search for alternative hosts. We cannot exclude the following theories: (i) a second generation onto new eggs laid by plant-leafhoppers able to develop on other plants (e.g., some Typhlocybinae species [93,94]), as already hypothesized for some egg parasitoids [57,95,96,97]; (ii) the specimens emerged in the second half of July could belong to a quiescent part of the overwintering population as observed in other specimens belonging to the family Thrichogrammatidae [98,99,100]. These hypotheses need to be deepened with further and targeted trials.

Only one specimen was observed to emerge from each egg. Therefore, the phenomenon of superparasitism in this species, meaning two or more holes per egg, was not observed like in other Oligositinae species [101,102] which would obviously have altered the parasitization rate. The females emerged in April, if males are present, begin to mate in a short time, on the contrary, if mating does not occur, immediately look for the host eggs into the bark to lay eggs. The oviposition behavior observed, is similar to other Trichogrammatidae species, showing antennal tapping on the host egg, before ovipositing, and their typical abdominal vibration [103,104].

Concerning the preliminary comparison of the parasitization rate between *O.* cf *collina* and *N. typhlocybae*, the dryinid parasitization rate detected ranged from 18 to 35% and was not too dissimilar to the values reported in other studies [31,105], while it was rather lower than those observed by other authors [27,106] who detected a parasitization over 70%. Our results could be explained with the possible reduction in the *N. typhlocybae* population caused by hyperparasitoids [65,107,108]. On the other hand, this could be attributed to the parasitization dynamics, which can oscillate over the years [80,81]. Nevertheless, it is well known that *N. typhlocybae* can perform a simultaneous predatory action in addition to the parasitization, which can have a significant impact on the flatid population [27,105]. Thus, we consider the parasitization rate observed in some sites, as a confirmation of the success of the dryinid settlement and its contribution to the control of the *M. pruinosa* population. However, the comparison of the parasitization rate performed by *O.* cf *collina* and *N. typhlocybae* pointed out a higher rate of the egg parasitoids. The aim of this research was to emphasize the egg parasitoid role in the flatid population control rather than comparing the efficiency in controlling *M. pruinosa* or underestimate that realized by the dryinid. Therefore, further in-depth studies are needed to quantify a possible synergic effect.

The Palaearctic origin of *O.* cf *collina* must be confirmed as well as the establishment of this new association in other regions of the Palaearctic area. In this regard, further studies are ongoing to identify the species and investigate its biological traits like voltinism and overwintering behavior.

## 5. Conclusions

In this study we describe a new association between the oophagous *O*. cf *collina* and the flatid *M. pruinosa*. During the two year study and in the two investigated areas, we found out a high rate of parasitization never detected before on *M. pruinosa*. The results show that the field parasitization rate increases with the host egg density. Furthermore, the interaction between host density and plant cover reveals how the parasitization rate increases along with the growth of the percentage of plants bearing the host eggs. This highlights that a minor habitat fragmentation can favour the egg search activity by the oophagous. Finally, the preliminary study concerning the comparison between the parasitization rate of *O*. cf *collina* and *N. typhlocybae* showed that in most of the surveyed sites the oophagous prevails on the dryinid.

The identification of an oophagous able to adapt to this flatid may deepen the knowledge on the planthopper containment effectiveness in some European countries, and open up new perspectives on its biological control. The evidence of a high biodiversity in the uncultivated areas within the vineyard agroecosystem, points out the need of further investigations to clarify their possible role as ecological compensation areas.

## Supplementary Materials

The following are available online at https://www.mdpi.com/2075-4450/11/9/610/s1, Figure S1: Map of the surveyed areas in Piedmont, Figure S2: Daily emergence of *Oligosita* cf *collina* from field collected parasitized eggs, Figure S3: Graphs of the residual diagnostic for the selected models concerning the probability of parasitization (A), and *Metcalfa pruinosa* oviposition rate (B), Table S1: Study on the parasitization rate of *Oligoista* cf *collina* during 2017 and 2018: coordinates of the collection sites, Table S2: Survey on the comparison between *Oligosita* cf *collina* and *Neodryinus typhlocybae*: coordinates for the collection sites relative to the comparison, Table S3: Results of the selected generalized linear mixed effects model (GLMM) testing the effects on the parasitization rate of Plant Species, Host Egg Density, Plant Coverage and the interaction between Host Egg Density and Plant Cover, Table S4: Candidate GLMM predicting the *Metcalfa pruinosa* egg parasitization (dependent variable correspond to success or failure of parasitization), Table S5: Results of the selected generalized linear mixed effects model (GLMM) testing the effects on the oviposition rate of Plant Species and Plant Coverage, Table S6: Candidate GLMM predicting the *Metcalfa pruinosa* oviposition (dependent variable corresponds to the count of laid eggs of *Metcalfa pruinosa*), Table S7: Parasitization rate of *Oligosita* cf *collina* and *Neodryinus typhlocybae* in each site.
