# Supplementary material for: Palaearctic Egg Parasitoids Interaction to Three Grapevine Exotic Pests in Northwestern Italy: A New Association Involving Metcalfa pruinosa
Source: Insects. 2020 Sep 8;11(9):610. doi: 10.3390/insects11090610 (PMC7564834; doi:10.3390/insects11090610)
Supplement: Supplementary file 1 [file insects-11-00610-s001.pdf]

Palaearctic egg parasitoids interaction to three grapevine exotic pests in northwestern Italy: a new association involving *Metcalfa pruinosa*

Federico Marco Bocca [1], Luca Picciau [1], Stefania Laudonia[2] and Alberto Alma [1,*]

[1] Department of Agricultural, Forest and Food Sciences and Technologies, University of Turin, Largo P. Braccini 2, 10095 Grugliasco (TO), Italy.
[2] Department of Agricultural Sciences, University of Naples Federico II, Via Università, 100, 80055 Portici (NA), Italy.
* Author to whom correspondence should be addressed; e-mail address: alberto.alma@unito.it; Tel: +39 011 6708534

Insects

Supplementary material

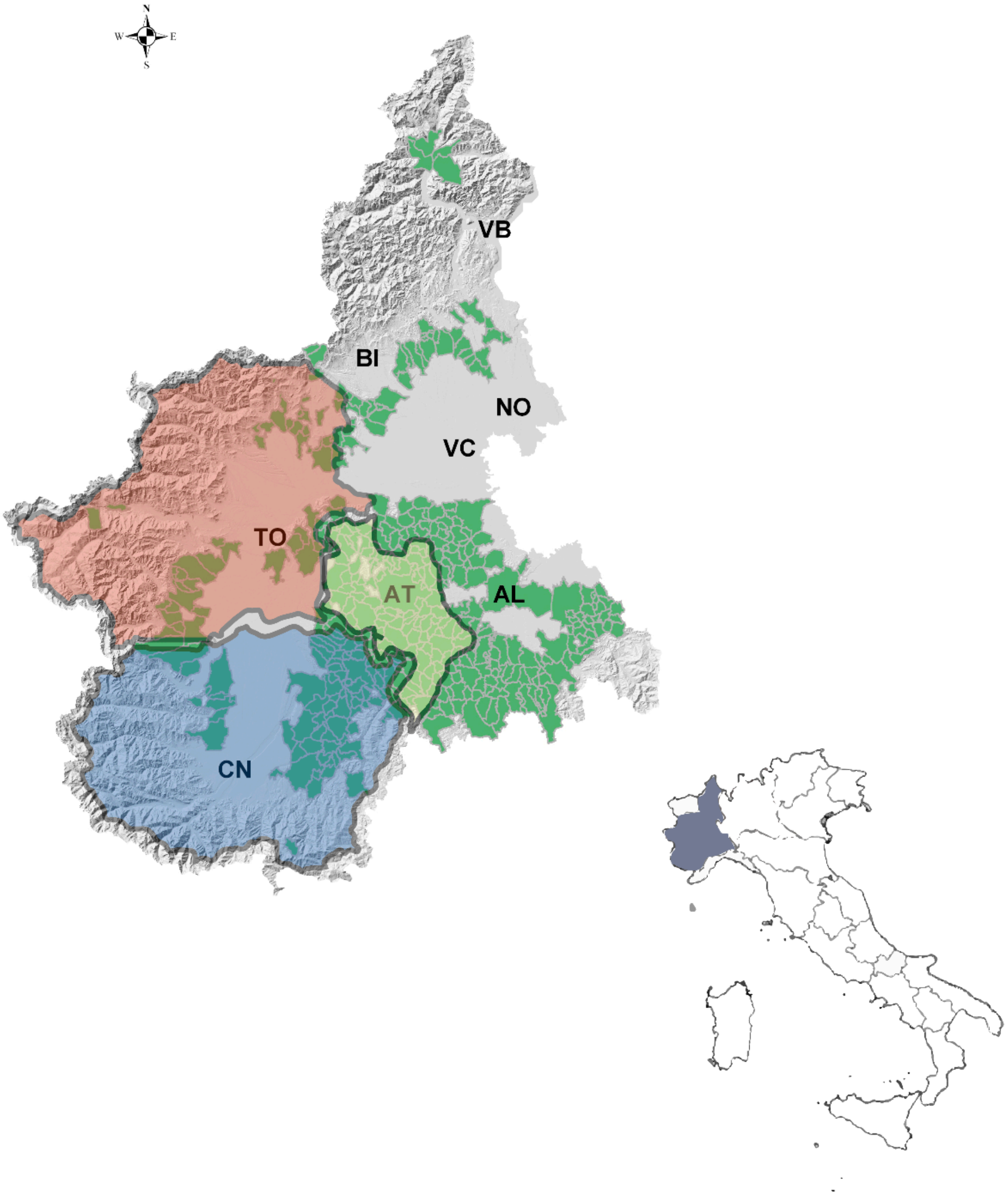

Figure S1. Map of the surveyed areas in Piedmont. Blue surface (Cuneo province), and yellow surface (Asti province) referred to the study on the parasitization rate of *Oligosita* cf *collina*. Red surface (Turin province) refers to the comparison between the parasitization rate of *Neodryinus typhlocybae* and *Oligosita* cf *collina* group on *Metcalfa pruinosa*. The green plots on the map represent the wine-growing municipalities in Piedmont region.

Table S1. Study on the parasitization rate of *Oligoista* cf *collina* during 2017 and 2018: coordinates of the collection sites.

Area	Year	Site	Coordinates
Asti Province	2017	1	44°49'15.4"N 8°18'04.5"E
		2	44°47'32.5"N 8°17'44.9"E
		3	45°03'07.2"N 8°01'59.4"E
		4	44°45'39.4"N 8°24'31.8"E
		5	44°52'04.4"N 8°18'51.6"E
	2018	6	44°57'43.5"N 8°15'28.6"E
		7	44°59'36.6"N 8°09'44.3"E
		8	44°57'42.9"N 8°17'09.5"E
		9	45°02'11.1"N 8°04'57.2"E
		10	44°49'17.3"N 8°01'08.7"E
Cuneo Province	2017	1	44°36′37.9′′N 8°12′24.7′′E
		2	44°36'50.6"N 8°11'51.4"E
		3	44°36'37.9"N 8°12'24.7"E

	4	44°48'26.6"N 7°58'55.1"E
	5	44°47'30.4"N 7°58'01.4"E
2018	6	44°30'42.9"N 7°56'47.6"E
	7	44°32'53.2"N 7°59'10.1"E
	8	44°41'31.8"N 8°14'35.2"E
	9	44°48'28.2"N 7°57'41.6"E
	10	44°42'29.4"N 8°19'22.6"E

Table S2. Survey on the comparison between *Oligosita* cf *collina* and *Neodryinus typhlocybae*: coordinates for the collection sites relative to the comparison.

Area	Year	Site	Coordinates
Turin Province	2018/2019		
		1	45°23'48.3"N 7°40'03.1"E
		2	45°18'41.6"N 7°54'16.5"E
		3	45°20'30.8"N 7°49'16.1"E
		4	45°17'27.5"N 7°56'30.1"E
		5	45°20'00.1"N 7°49'53.8"E
		6	45°19'34.4"N 7°51'18.2"E
		7	45°16'43.3"N 7°37'56.9"E
		8	45°25'59.4"N 7°57'22.4"E

Table S3. Results of the selected generalized linear mixed effects model (GLMM) testing the effects on the parasitization rate of Plant Species, Host Egg Density, Plant Cover and the interaction between Host Egg Density and Plant Cover.

**Parameter**	**Estimate**	**SE**	**z**	**P**
(Intercept)	-1.08589	0.08678	-12.512	***
Corylus avellana.	-0.28625	0.09458	-3.026	**
Vitis spp.	0.26396	0.06839	3.859	***
Host Egg Density	0.52363	0.06935	7.550	***
Plant Cover	0.28382	0.05300	5.355	***
Host Egg Density: Plant Cover	0.10732	0.05420	1.980	0.047704

SE standard error of parameter estimates. z z-score testing whether the parameter estimate is significantly different from zero. Pr(|z|) probability of the observed z-score being greater than the critical value. Significant at ≤ 0.05 (*). ≤ 0.01 (**). ≤ 0.001(***). not significant (NS).

Table S4. Candidate GLMM predicting the *Metcalfa pruinosa* egg parasitization (dependent variable corresponds to success or failure of parasitization). Best subset selection approach was used; results shown only the top 10 candidate models. Group Site nested within Area was included as random effect in all models to take into accounts the dependences among measures in each Area (1| area/site)".

	**Response variable: number of eggs ejected from communal nest**			**Overall model significance**	
	**Fixed effects**	**AIC$_i$**	**ΔAIC$_i$**	**Wald's X^2**	***P***
[1.]	~plant species+plant cover+plant species:host eggs density+host eggs density:plant cover	410.626317189967	0.000000	725.3	<0.0001
[2.]	~plant species+host eggs density+plant cover+plant species:host eggs density+host eggs density:plant cover	410.626320914904	0.000004	725.3	<0.0001
[3.]	~plant species+plant cover+plant species:host eggs density	411.497259738122	0.870943	722.43	<0.0001
[4.]	~plant species+host eggs density+plant cover+plant species:host eggs density	411.497259870215	0.870943	722.43	<0.0001
[5.]	~plant species+host eggs density+plant cover+host eggs density:plant cover	411.695475559671	1.069158	720.23	<0.0001
[6.]	~plant species+host eggs density+plant cover+plant species:host eggs density+plant cover:plant species+host eggs density:plant cover	411.720921207443	1.094604	728.21	<0.0001
[7.]	~plant species+plant cover+plant species:host eggs density+plant cover:plant species+host eggs density:plant cover	411.720921876676	1.094605	728.21	<0.0001
[8.]	~plant species+host eggs density+plant species:host eggs density+plant cover:plant species+host eggs density:plant cover	411.720922147976	1.094605	728.21	<0.0001

[9.]	~plant species+plant species:host eggs density+plant cover:plant species+host eggs density:plant cover	411.720923594172	1.094606	728.21	<0.0001
[10.]	~plant species+plant cover+plant species:host eggs density+plant cover:plant species	412.065634384171	1.439317	725.86	<0.0001

Table S5. Results of the selected generalized linear mixed effects model (GLMM) testing the effects on the oviposition rate of Plant Species and Plant Cover.

**Parameter**	**Estimate**	**SE**	***z***	**P**
(Intercept)	2.20649	0.07570	29.147	***
Corylus avellana	-0.36801	0.10752	-3.423	***
Vitis spp.	0.27576	0.10049	2.744	**
Plant Cover	0.22721	0.04606	4.933	***

SE standard error of parameter estimates. *z* z-score testing whether the parameter estimate is significantly different from zero. Pr(|z|) probability of the observed z-score being greater than the critical value. Significant at ≤ 0.05 (*). ≤ 0.01 (**). ≤ 0.001(***). not significant (NS).

Table S6. Candidate GLMM predicting the *Metcalfa pruinosa* oviposition (dependent variable corresponds to the count of laid eggs of *Metcalfa pruinosa*). Best subset selection approach was used; results shown only the top 10 candidate models. Group Site nested within Area was included as random effect in all models to take into accounts the dependences among measures in each Area (1| area/site)".

	**Response variable: number of eggs ejected from communal nest**			**Overall model significance**	
	**Fixed effects**	**AIC$_i$**	**ΔAIC$_i$**	**Wald's X^2**	***P***
[1.]	~plant species+plant cover+ offset(log(plant))	605.161772115018	0.000000	591.16	<0.0001
[2.]	~plant species+plant cover:plant species+ offset(log(plant))	605.794000229603	0.632228	587.79	<0.0001
[3.]	~plant species+plant cover+plant cover:plant species+ offset(log(plant))"	605.794000232042	0.632228	587.79	<0.0001
[4.]	~plant species+plant cover+year+ offset(log(plant))	606.818019074198	1.656247	590.82	<0.0001
[5.]	~plant species+year+plant cover:plant species+ offset(log(plant))	607.543683592333	2.381911	587.54	<0.0001
[6.]	~plant species+plant cover+year+plant cover:plant species+ offset(log(plant))	607.543683601835	2.381911	587.54	<0.0001
[7.]	~plant cover+plant species:year+ offset(log(plant)	607.924954709873	2.763183	587.92	<0.0001
[8.]	~plant cover+year+plant species:year+ offset(log(plant))	607.924954710409	2.763183	587.92	<0.0001
[9.]	~plant species+plant cover+plant species:year+ offset(log(plant))	607.924954713611	2.763183	587.92	<0.0001
[10.]	~plant species+plant cover+year+plant species:year+ offset(log(plant))	607.924954714603	2.763183	587.92	<0.0001

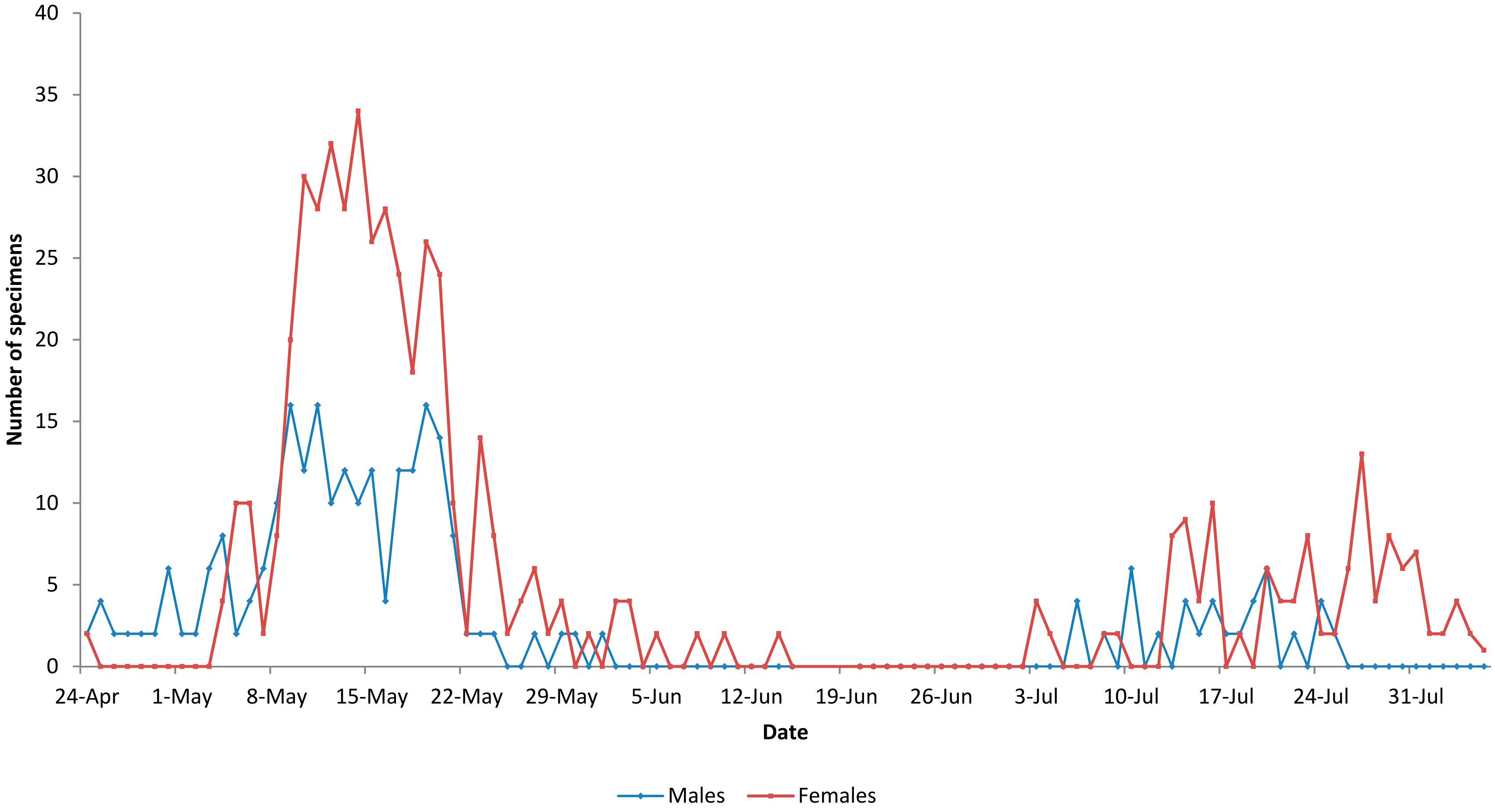

Figure S2. Daily emergence of *Oligosita* cf *collina* from field collected parasitized eggs.

Table S7. Parasitization rate of *Oligosita* cf *collina* and *Neodryinus typhlocybae* in each site.

**Site**	**Parasitization rate of *Oligosita* cf *collina***	**Parasitization rate of *Neodryinus typhlocybae***
1	0.34	0.30
2	0.44	0.25
3	0.47	0.18
4	0.36	0.20
5	0.45	0.32
6	0.41	0.26
7	0.39	0.29
8	0.46	0.27

Figure S3. Graphs of the residual diagnostic for the selected models concerning the probability of parasitization **(A)**, and *Metcalfa pruinosa* oviposition rate **(B)**.

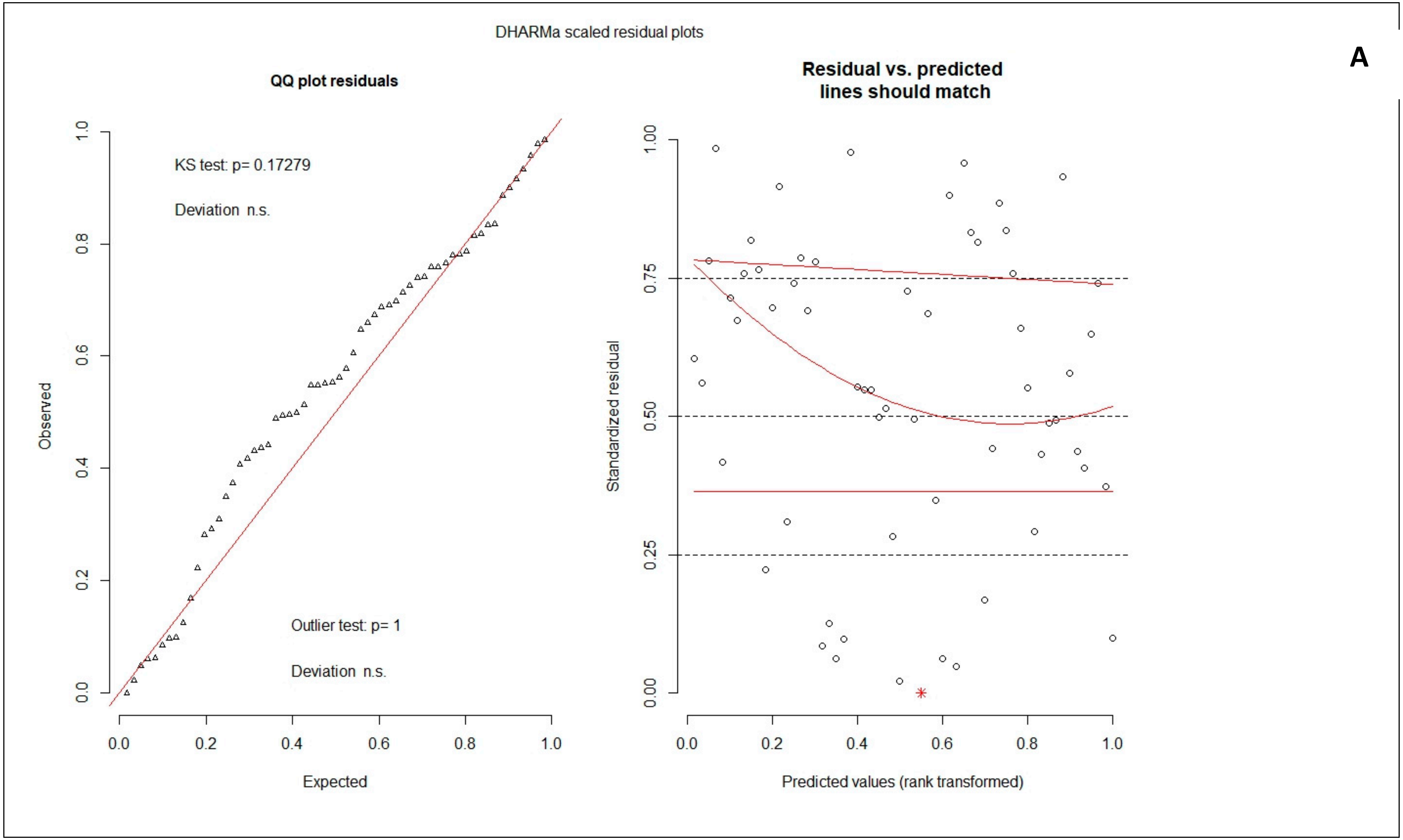

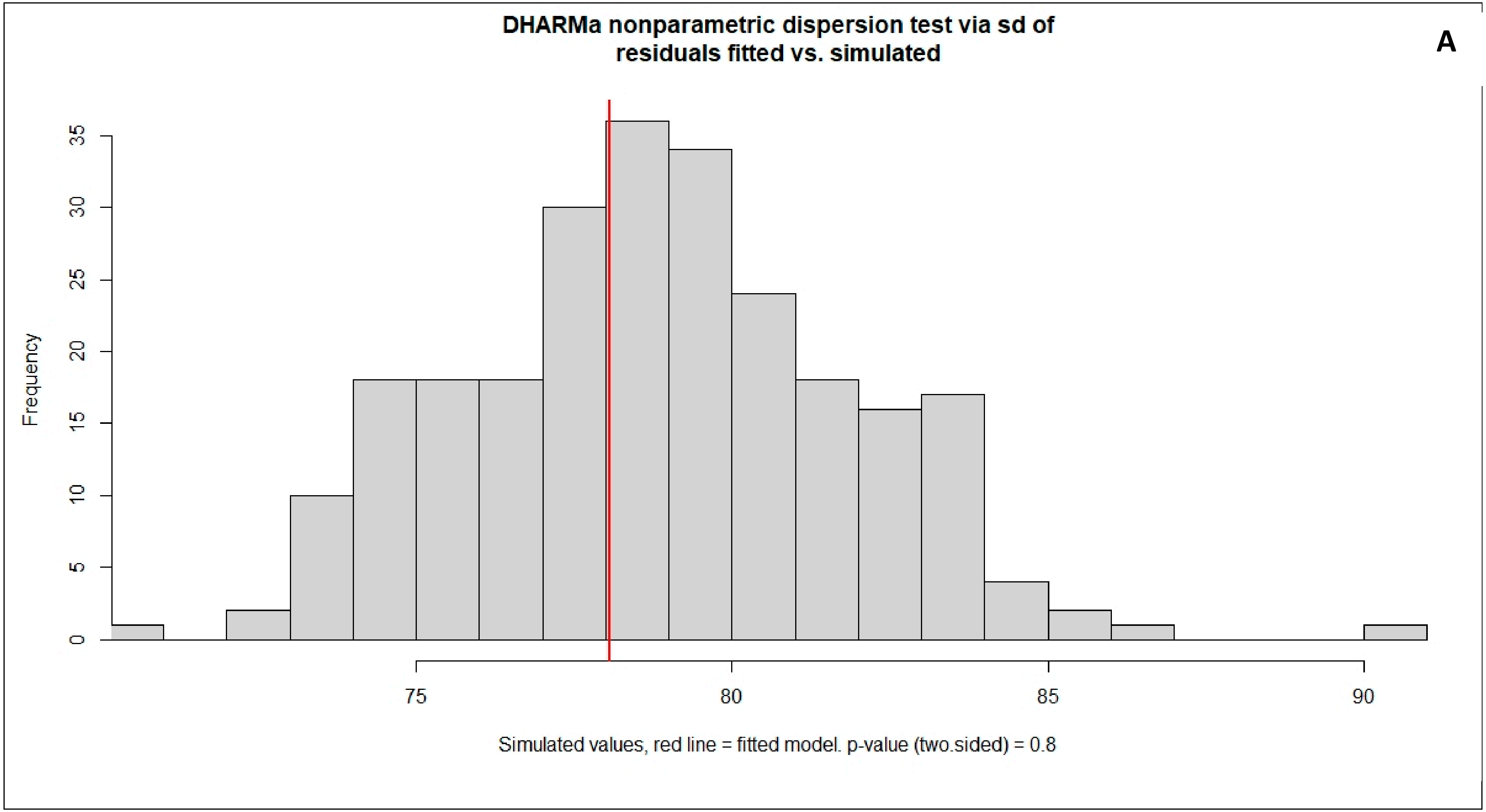
DHARMa nonparametric dispersion test via sd of
residuals fitted vs. simulated
A
Frequency
0
5
10
15
20
25
30
35
75
80
85
90
Simulated values, red line = fitted model. p-value (two.sided) = 0.8

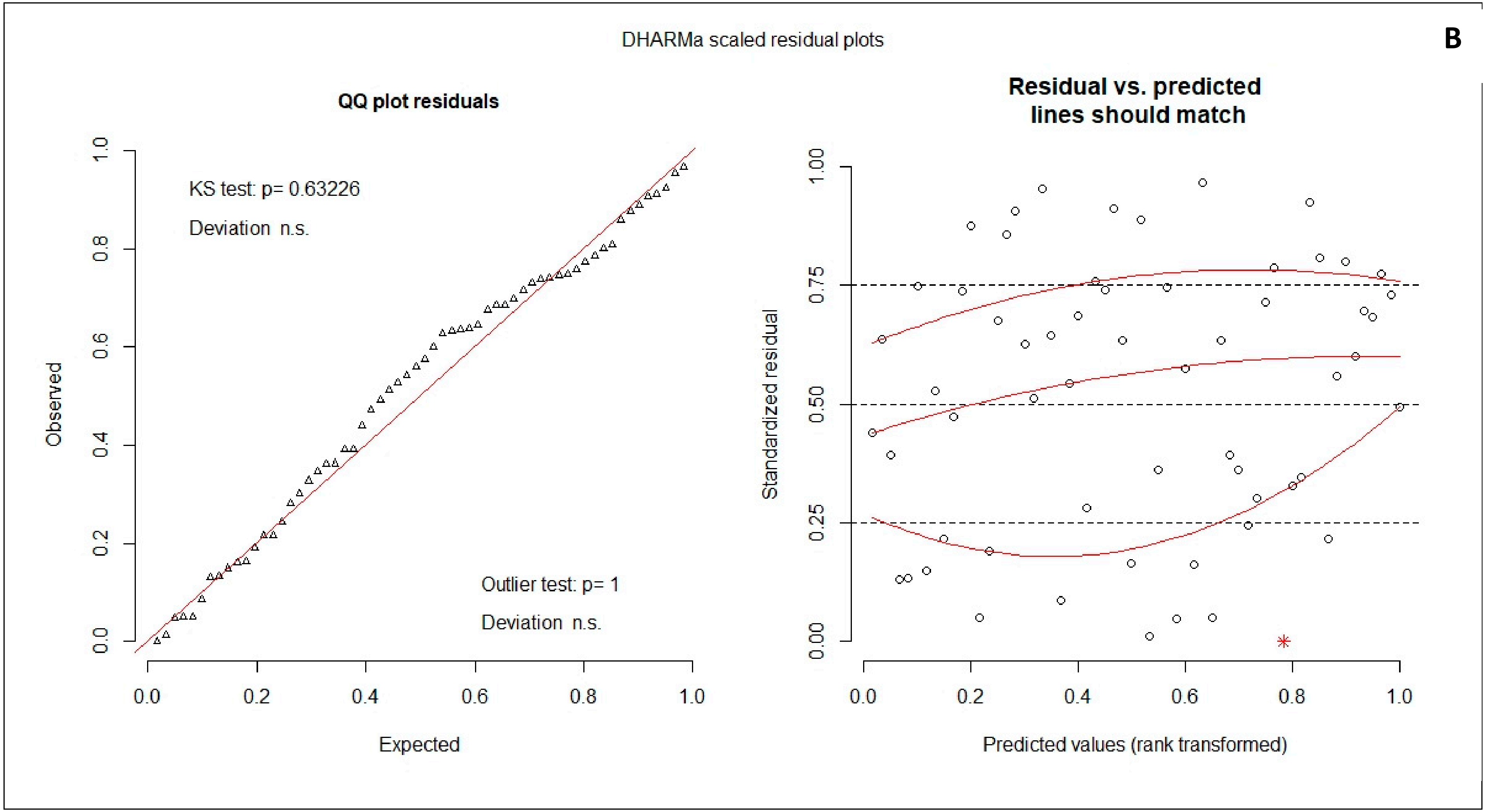

DHARMa scaled residual plots
B
QQ plot residuals
KS test: p= 0.63226
Deviation n.s.
Outlier test: p= 1
Deviation n.s.
Observed
Expected
0.0
0.2
0.4
0.6
0.8
1.0
Residual vs. predicted
lines should match
Standardized residual
Predicted values (rank transformed)
0.00
0.25
0.50
0.75
1.00

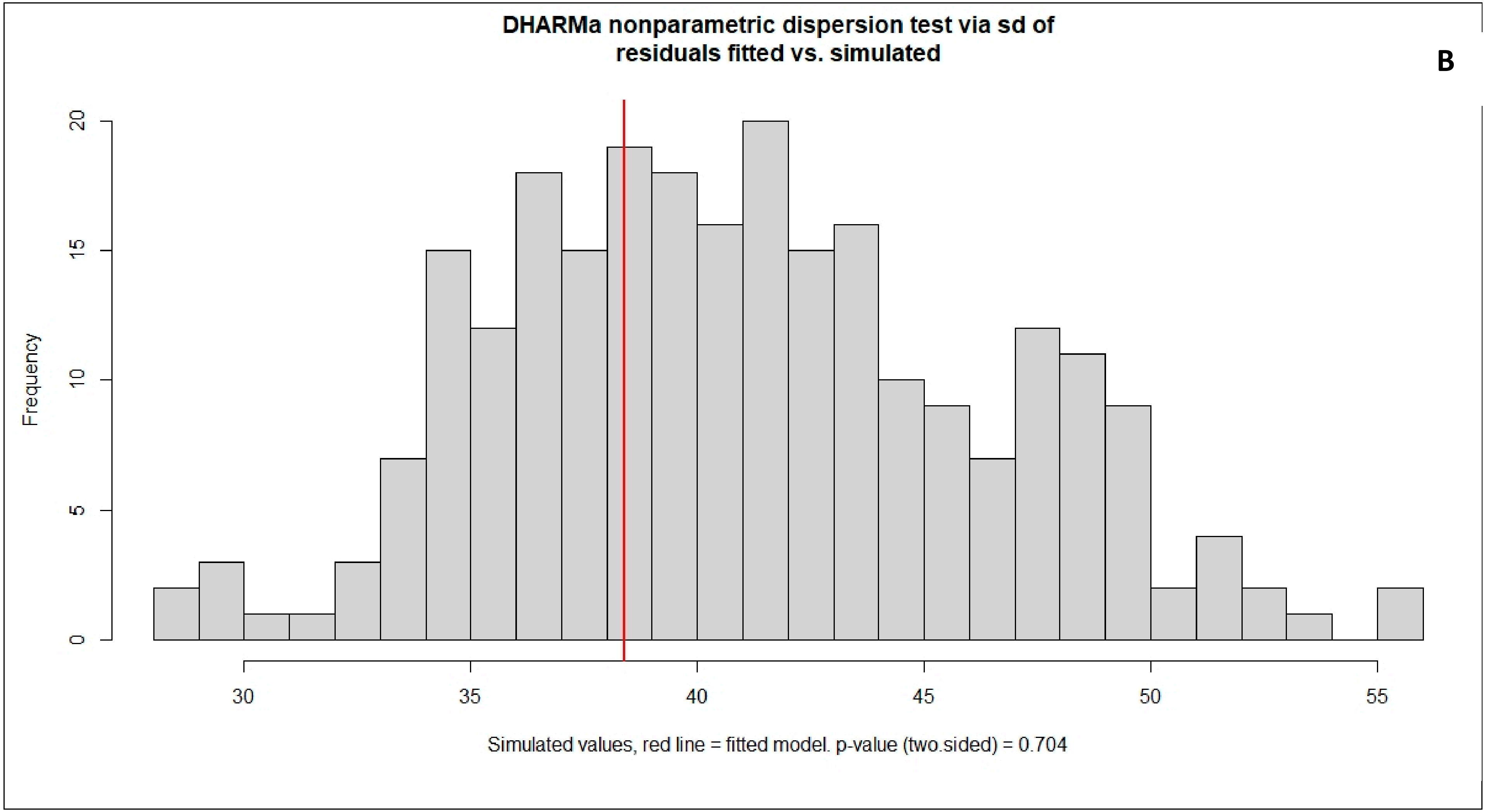

DHARMa nonparametric dispersion test via sd of
residuals fitted vs. simulated
B
Frequency
0
5
10
15
20
30
35
40
45
50
55
Simulated values, red line = fitted model. p-value (two.sided) = 0.704
